# Effects of Mesenchymal Stem Cell Coculture on Human Lung Small Airway Epithelial Cells

**DOI:** 10.1155/2020/9847579

**Published:** 2020-03-27

**Authors:** Eva Schmelzer, Vitale Miceli, Cinzia Maria Chinnici, Alessandro Bertani, Jörg C. Gerlach

**Affiliations:** ^1^Department of Surgery, University of Pittsburgh, Pennsylvania, USA; ^2^Research Department, IRCCS-ISMETT Istituto Mediterraneo per i Trapianti e Terapie ad Alta Specializzazione, UPMC Italy, Palermo, Italy; ^3^Fondazione Ri.MED, Regenerative Medicine and Biomedical Technologies Unit, UPMC Italy, Palermo, Italy; ^4^Regenerative Medicine and Biomedical Technologies Unit, IRCCS-ISMETT Palermo, Italy; ^5^Division of Thoracic Surgery and Lung Transplantation, IRCCS-ISMETT Palermo, Italy; ^6^Department of Bioengineering, University of Pittsburgh, Pennsylvania, USA

## Abstract

Mesenchymal stem cells (MSCs) and their secreted extracellular vesicles have been used effectively in different lung disease animal models and clinical trials. Their specific beneficial effects, the potential differences between MSCs derived from different organs, and interactions between MSC products and target cells still need to be studied further. Therefore, we investigated the effects of secreted products of human MSCs derived from the bone marrow and adipose tissue on human lung small airway epithelial (AE) cells *in vitro*. AE cells were cocultured with MSCs in inserts that allowed the free exchange of medium but did not allow direct cell-to-cell contact. We examined the effects on AE cell viability, proliferation, cell numbers, expression of AE cell-specific genes, and CD54 (intercellular adhesion molecule 1 (ICAM1)) surface positivity, as well as the secretion/uptake of growth factors relevant for AE cell. We found that coculture increased the viability of AE cells. The majority of AE cells expressed CD54 on their surface, but the percentage of cells being positive for CD54 did not increase in coculture. However, ICAM1 gene expression was increased in coculture. Also, we observed increased gene expression of mucin (MUC1), a lung-enriched cell surface glycoprotein. These observed effects were the same between bone marrow and adipose tissue MSCs. However, MSCs derived from adipose tissue reduced angiopoietin concentrations in coculture, whereas those from the bone marrow did not. Conclusively, MSCs influenced AE cells positively by increasing their viability and affecting gene expression, with some effects being specific for the tissue origin of MSCs.

## 1. Introduction

Airway epithelial (AE) cells are the major component of the airway epithelial sheet, forming a protective barrier along with other cell types such as endothelial and mesenchymal cells (MSCs) [1]. The epithelial sheet, however, does not only provide air exchange and presents a physical barrier but also has multiple biochemical functions. These comprise mucus and fluid secretion and clearance, secretion of mediators, clearance of inhaled substances, activation of inflammatory cells, and more [2]. AE cells are involved in several diseases such as asthma, chronic obstructive pulmonary disease, and bronchogenic carcinoma; a minimum of eight distinct epithelial cell types has been identified that have been classified into basal, secretory, and ciliated types [3–5].

In different lung disease animal models and clinical trials, MSCs have been transplanted effectively (for a review summarizing models and clinical trials in chronic obstructive pulmonary disease, see [6]). Positive effects of the transplanted MSCs on pathological conditions have been observed and were attributed to cellular fusion with host cells as well as the release of numerous proteins, cytokines, extracellular vesicles (EVs), and RNA. By implementing cell-free therapies, the sole use of EVs (including exosomes and microvesicles) derived from MSCs has resulted in positive therapeutic effects, as summarized in [7, 8]. The diverse conditions that have been treated with EVs in animal studies included pulmonary hypertension [9, 10] and endotoxin-induced pneumonia [11, 12]. The clearance of alveolar fluid from human lungs in an *ex vivo* model was increased by EVs derived from human mesenchymal stem cells, and these had the same effects as the MSCs themselves in restoring human donor lungs [13]. In silica-induced pulmonary fibrosis, transplanted MSCs successfully integrated and differentiated into type II lung cells. Although the therapeutic effects of the transplanted cells were superior to that of microvesicle treatment alone, the microvesicle treatment also significantly reduced the symptoms of idiopathic pulmonary fibrosis [14].

Based on the positive outcomes in transplantation and vesicle treatment *in vivo* studies, we were interested in investigating the specific effects of MSCs on AE cells *in vitro*. We chose MSCs derived from two different origins, i.e., the bone marrow and adipose tissue. For potential clinical applications, these tissues are two of the easiest accessible autologous sources that are available in larger quantities. We were explicitly interested if differences between these two sources exist in their secretions and effects on AE cells. Therefore, MSCs were cocultured in inserts that allowed the free exchange of medium but did not allow direct contact with AE cells. We examined the effects of secreted substances of MSCs on AE cell proliferation, cell numbers, gene expression, cell viability, CD54 positivity, and the secretion/uptake of AE cell-specific growth factors.

## 2. Material and Methods

### 2.1. Cell Culture

Cryopreserved primary human small airway epithelial (AE) cells, as well as MSCs from adipose (AD) and bone marrow (BM) tissues from three different donors each, were obtained from various companies (ScienCell, Carlsbad, CA; ATCC, Manassas, VA; Lifeline Cell Technology, Frederick, MD; and Lonza, Walkersville, MD). Before the actual experiments, we cultured both cryopreserved cell types separately for expansion of cell numbers and adaption to culture. After thawing, both cell types were seeded at a density of 5,000 cells per cm^2^ in cell culture flasks. Cell viabilities and numbers were monitored by trypan blue exclusion in a Neubauer chamber. AE cells were cultured in an airway epithelial basal medium plus supplement kit (ATCC) and 100 units/mL penicillin, 100 *μ*g/mL streptomycin, and 0.25 *μ*g/mL fungizone (Gibco/Thermo Fisher Scientific, Pittsburgh, PA). MSCs were cultured in DMEM low glucose, containing Glutamax and supplemented with 10% FBS and 100 units/mL penicillin, 100 *μ*g/mL streptomycin, and 0.25 *μ*g/mL fungizone (all Gibco/Thermo Fisher Scientific). Both cell types were used for the actual coculture experiments after the first passage.

For the coculture experiments, AE cells were placed into the bottom wells of transwell permeable 6-well plate supports (Corning, Corning, NY) at a density of 10,000 cells per cm^2^, resulting in 96,000 cells per well. After 24 h, MSCs were seeded in the inserts of the same wells. In order to avoid the occurrence of confluency of MSCs during the three-day culture period, cells were seeded at a lower density of 5,000 cells per cm^2^, resulting in 23,350 cells per insert. Each of the AE cells derived from three different donors was cocultured with three different donor-derived BM or AD mesenchymal stem cells. Inserts had a pore size of 0.4 *μ*m, permitting the free exchange of molecules but preventing cell migration or contact. Controls included cultures of AE cells without addition of mesenchymal stem cells and cultures of MSCs without AE cells and medium without the addition of any cell type. All cells were cultured in a supplemented airway epithelial basal medium. After three days in coculture, AE cells and medium were collected for analyses. Cells were monitored during culture using microscopy, and images were acquired with a phase-contrast light microscope (Invertoskop C), equipped with a camera (AxioCam MRc) and software (AxioVision Vs40, V4.2.0.0) (Zeiss, Thornwood, NY).

### 2.2. Flow Cytometry

Cultured AE cells were detached with 0.05% trypsin-EDTA (ATCC), and enzymatic action was stopped by adding 5% FBS in PBS (Gibco/Thermo Fisher Scientific). Cells were washed with PBS and stained with eBioscience Fixable Viability Dye eFluor 780 (Invitrogen/Thermo Fisher Scientific). Subsequently, cells were stained for 15 min at 4°C with mouse IgG1 anti-human CD54-AF647 or corresponding isotype control (BioLegend, San Diego, CA) in buffer containing 0.5% bovine serum albumin (Sigma-Aldrich, St. Louis, MO), 2 mM disodium EDTA (Sigma-Aldrich) in PBS (without calcium and magnesium (Gibco/Thermo Fisher Scientific)), and 10% human FcR blocking reagent (Miltenyi Biotec, Bergisch Gladbach, Germany). For costaining of nuclear proliferation marker Ki67, cells were fixed in ice-cold 70% ethanol, including 1% FBS. Cells were fixed and permeabilized overnight at -20°C. After washing with buffer, cells were incubated with mouse IgG1 anti-human Ki67-PE antibody or isotype control (BD Biosciences, San Jose, CA) for 30 min at room temperature in the dark. Cells were analyzed in a FACSAria II flow cytometer (BD Biosciences). Controls included nonstained cells and cells incubated with corresponding isotype controls. Compensation beads (eBioscience/Thermo Fisher Scientific) were used to compensate potential spectral fluorochrome overlap; compensation for the eFluor 780 dye was done with an equal mix of live and dead AE cells (dead cells were obtained by heating cell suspensions at 65°C for 1 min and cooling on ice for 1 min). Raw data were analyzed using FlowJo software version 10.4.1 (FlowJo, Ashland, OR). Cell debris and cell doublets were excluded by applying an initial forward versus side scatter gate.

### 2.3. Gene Expression Analyses

Cells in culture were lysed directly with RLT buffer containing 1% beta-mercaptoethanol (Sigma-Aldrich). Nucleic acids were isolated using shredder and isolation columns (AllPrep DNA/RNA Mini Kit (Qiagen, Hilden, Germany)). Potential traces of DNA on RNA extraction columns were digested by DNase treatment directly on columns. Concentrations of nucleic acids were determined using Quant-iT Assay kits and a Qubit fluorometer (Invitrogen/Thermo Fisher Scientific). RNA was reverse transcribed to cDNA with the High-Capacity cDNA Reverse Transcription Kit (Applied Biosystems, Carlsbad, CA). Gene expression was analyzed using real-time PCR (StepOnePlus system and software version 2.0) and predesigned TaqMan probe and primer assay mixes for intercellular adhesion molecule 1 (ICAM1), aquaporin 5 (AQP5), mucin 1 (MUC1), secretoglobin family 1A member 1 (SCGB1A1), tubulin alpha-1A (TUBA1A), and gap junction alpha-1 protein (GJA1) with a gene expression master mix (Applied Biosystems). Peptidylprolyl isomerase A (PPIA), also known as cyclophilin A, was used as the housekeeping gene for endogenous normalization, as it has been recommended by He et al. [15] to be the most suitable one compared to other normally used housekeeping genes such as beta-actin or glyceraldehyde-3-phosphate dehydrogenase. Gene expression was quantified using the ddCt method. As a positive control, cDNA that was reverse transcribed from DNase-treated RNA of human adult lung tissue (BioChain, Newark, CA) was implemented. No template (water) was used as a negative control. Each of the samples was analyzed with two technical repeats.

### 2.4. Enzyme-Linked Immunosorbent Assays

The secretion of coagulation factor 3 and of growth factor angiopoietin-2 into cell culture media was determined using enzyme-linked immunosorbent assays (ELISAs) (RayBiotech, Norcross, GA). The OD at 450 nm of samples was read using a Synergy H1 hybrid multimode microplate reader and Gen5 data analysis software (BioTek, Winooski, VT). The medium was used as blank. Every sample was analyzed with two technical repeats.

### 2.5. Lactate Dehydrogenase Activity

The viability of cells in culture was determined by detecting the release of LDH enzyme in cell culture medium samples. LDH is only released by damaged cells; thus, an increased enzyme activity correlates negatively with cell viability. Enzyme activity was measured using the QuantiChrom LDH Kit (BioAssay Systems, Hayward, CA) according to the manufacturer's instruction. The optical density (OD) at 565 nm of samples was read immediately and after 25 min, using a Synergy H1 hybrid multimode microplate reader and Gen5 data analysis software (BioTek, Winooski, VT). LDH activity was calculated as IU/L. Water was used as a negative control, and culture medium was used as blank. Each of the samples was analyzed with two technical repeats.

## 3. Results

### 3.1. Cell Culture and Growth

**Figure 1:**
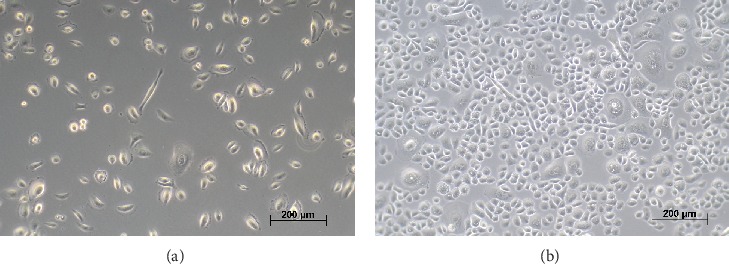
Small airway epithelial cells in culture. Small airway epithelial cells are well-attached one day after plating (a) and had formed a near confluent monolayer after four days of culture (b). Phase microscopy, scale bars 200 *μ*m.

One day after plating, AE cells were attached well to the cell culture surface and had formed a nearly confluent monolayer at harvest on the fourth day (Figure 1). AE cells had a population doubling time of 40.6 ± 2.0 hours.

**Figure 2:**
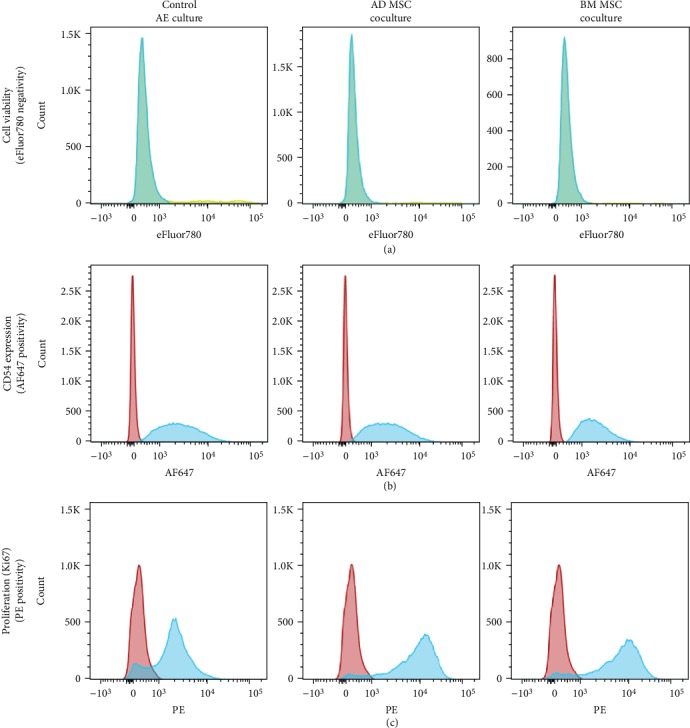
Flow cytometry of small airway epithelial cells. Representative analyses of small airway epithelial (AE) cells investigated in flow cytometry. AE cells were either cultured alone without any addition of mesenchymal cells (MSC), which are the control cultures, left, or cocultured with MSCs derived from either adipose tissue (AD), middle, or bone marrow (BM), right. (a) Determination of cell viability using the eFluor 780 viability dye, which stains exclusively dead cell positive (yellow) compared to unstained controls (blue). (b) Detection of CD54-positive cells (blue), with isotype controls (red). (c) Detection of proliferating Ki67-positive cells (blue), with isotype controls (red).

In coculture with mesenchymal stem cells, the proliferation rates of AE cells were slightly increased compared to controls, although not statistically significant. As measured by Ki67 positivity in flow cytometry (see Figure 2(c) for example plots), 76.4 ± 4.4% of AE cells in culture without the addition of mesenchymal cells were proliferating. The addition of mesenchymal cells led to an increase in Ki67 positivity to 80.7 ± 5.0% and 80.3 ± 5.6% when AE cells were cocultured with BM or AD cells, respectively.

Both types of mesenchymal cells were characterized in-depth in a previous publication [16], which confirmed their expression of typical mesenchymal surface markers such as CD90 and CD73, the absence of hematopoietic markers such as CD34 and CD45, their multilineage differentiation potential, and the secretion of growth factors and cytokines.

Total AE cell numbers were not affected by coculture. At harvest day 4, AE cell numbers were 51,429 ± 25,061 cells/cm^2^ and remained about the same with 50,714 ± 18,214 cells/cm^2^ and 55,357 ± 27,857 cells/cm^2^ in coculture with BM or AD cells, respectively.

### 3.2. Cell Viability

The addition of MSCs significantly increased the viability of AE cells in culture. After three days of culture, AE cells alone had a mean viability of 85.8 ± 4.6%, as measured by eFluor 780 labeling in flow cytometry (see Figure 2(a) for example plots). The addition of BM MSCs increased AE cell viability to 92.6 ± 1.9%, and coculture with AD MSCs increased AE cell viability to 92.8 ± 1.0%, both being significantly different to controls (*p* < 0.05). Furthermore, the LDH activity of 2.3 ± 0.8 IU/L of control cultures was reduced in coculture with mesenchymal stem cells. The addition of BM or AD MSCs decreased LDH activity to 1.7 ± 1.0 IU/L and 1.1 ± 0.3 IU/L, respectively.

### 3.3. Flow Cytometry for CD54

CD54, or intercellular adhesion molecule 1 (ICAM1), is a major surface glycoprotein of AE cells. It is the main rhinovirus adhesion site of the respiratory tract [17] and can be induced by various stimuli, including cytokines [18] and cigarette smoke [19]. Further, it is a member of the immunoglobulin superfamily, a ligand for lymphocytes, and regulates inflammation [20, 21]. Based on its essential role in healthy and pathological conditions of the airway epithelium, we investigated if the expression of surface CD54 of AE cells was influenced by the presence of BM or AD mesenchymal stem cells. We found that the majority of AE cells (91.5 ± 11.4%) were CD54 positive (see Figure 2(b) for example plots). Neither the coculture with BM nor AD MSCs changed significantly the expression of CD54, which were determined as 89.3 ± 14.8% and 91.2 ± 10.5%, respectively.

### 3.4. Gene Expression

We analyzed the effects of MSCs on the expression of genes typical for specific AE cell types and functions (Table 1). Genes included aquaporin 5 (AQP5), gap junction alpha-1 protein (GJA1), mucin 1 (MUC1), secretoglobin family 1A member 1 (SCGB1A1), tubulin alpha-1A (TUBA1A), and intercellular adhesion molecule 1 (ICAM1). The water channel protein AQP5 is of major importance for transporting water from the airspace to the capillary bed [22, 23]. GJA1, also known as connexin 43, is a frequent gap junction protein found in most lung cell types but is the exclusive gap junction protein of alveolar macrophages [24]. The membrane-bound MUC1 is a mucin enriched specifically in the small airway epithelium [25]. The low-molecular-weight secretoglobin SCGB1A1 is expressed and secreted by club cells [26], and the ciliated small airway epithelium strongly expresses TUBA1A [25].

While we investigated CD54 expression on the surface by flow cytometry and did not see effects of mesenchymal cells, we also investigated its gene expression, as its expression is mainly regulated at the transcriptional level [27]. The coculture with both types of MSCs increased the expression of ICAM1 and MUC1 significantly, about twofold and fourfold, respectively, when compared to controls. Although the expression of other analyzed genes was also increased, these differences were not statistically significant.

### 3.5. Protein Secretion

We investigated the secretion and uptake of proteins relevant for AE cell types into the cell culture medium and were interested if MSCs influence AE cell protein secretion or uptake. We measured human coagulation factor 3 and angiopoietin 2 in cell culture media using ELISAs (Table 2). Coagulation factor 3, or tissue factor, is the initiator of the coagulation cascade. AE cells have been shown to release coagulation factor 3 in response to proinflammatory stimuli [28], which decreases wound-healing time and improves the recovery of inflammation. Angiopoietin 2 is mostly expressed by endothelial cells and has been shown to be a mediator of necrosis in lung hyperoxia [29].

AE cells were cultured for three days either alone or in coculture with MSCs in inserts. Concentrations were measured in culture medium that had no addition of any cells (medium blank) and were used subsequently as blank controls and subtracted from all other measurements. Net protein concentrations of AE cell in coculture were calculated by subtracting concentrations of mesenchymal stem cell cultures from those of cocultures.

In cell culture medium alone, we could only detect negligible traces of coagulation factor 3. Both types of MSCs did not secrete or took up substantial amounts of coagulation factor 3. AE cells alone secreted considerable amounts of coagulation factor 3 (600.5 ± 182.6 pg/mL) into the medium. The addition of either mesenchymal stem cell type did not influence significantly the secretion or uptake of coagulation factor 3 of AE cells.

We measured that cell culture medium *per se* contained 51.9 ± 0.8 pg/mL angiopoietin 2. We found that both mesenchymal cell types secreted or took up only minimal amounts of angiopoietin 2 from the cell culture medium. When AE cells were cultured alone, they took up 15.8 ± 4.7 pg/mL angiopoietin 2 from the cell culture. The addition of BM mesenchymal cells in coculture did not change the uptake of AE cells of angiopoietin 2 significantly. The coculture with AD mesenchymal cells, however, reduced significantly the uptake of AE cells of angiopoietin 2 from the cell culture medium to nearly zero (1.0 ± 11.2 pg/mL).

## 4. Discussion

Commonly, MSCs of various organs have been considered to possess equal properties, including profiles of surface markers, gene expression, and protein secretion. More recent data, however, have revealed their distinctive characteristics. For example, exosomes derived from adipose MSCs had much higher enzyme (neprilysin) levels than those from bone marrow MSCs [30]. In our previous research, we had observed that MSCs isolated from six different human tissues demonstrated differences in their expression of lineage-specific genes, the secretion of growth factors, multilineage differentiation potential, and surface markers including CD105, CD45, and CD34 [16].

Here, we compared MSCs derived from the bone marrow and adipose tissue for their capacity to influence small AE cells. We found that both mesenchymal stem cell types influenced AE cells positively by increasing their viability and affecting gene expression, with some effects being specific for the tissue origin of mesenchymal stem cells. In particular, we observed that MSCs derived from AD reduced angiopoietin-2 concentrations in coculture, whereas those from BM did not. Secreted products from MSCs can be of important therapeutic value in various pulmonary diseases (for review, see [31]). A beneficial effect of inhibition of angiopoietin-2 has been investigated for cardiac transplantation [32], preventing transplant ischemia-reperfusion injury and chronic rejection. Interestingly, MSCs respond to stress signals from diseased tissue by secreting numerous substances. Among these, angiogenic growth factors, such as angiopoietin-1 and vascular endothelial growth factor, are of high relevance in stimulating vascular regeneration. In our previous studies, we could detect substantial secretion of several paracrine factors that are of relevance for angiogenesis, including vascular endothelial growth factor and hepatocyte growth factor, from human placenta-derived and amnion-derived MSCs [33, 34]. By using a hypoxia induction, exosomes from adipose tissue-derived MSCs supported angiogenesis in an *in vivo* mouse model [35]. The levels of several angiogenic growth factors, including vascular endothelial growth factor, were significantly higher in the hypoxia-induced MSCs than in controls. The enhanced secretion of angiopoietin-1 and keratinocyte growth factor of MSCs from the bone marrow was also shown to lower inflammation and apoptosis in alveolar epithelial cells that had been stimulated with lipopolysaccharides [36]. EVs from bone marrow MSCs also increased angiopoietin-1 secretion of lung microvascular endothelial cells and thereby restored their protein permeability [37].

Compared to angiopoietin-1, few studies have investigated the role of angiopoietin-2 in mesenchymal stem cell-related outcomes. An induction of angiopoietin-2 and angiopoietin-1 in mouse host hippocampus cells was observed when donor bone marrow-derived human MSCs were transplanted in a model of cerebral ischemia and reperfusion [38], indicating an indirect effect on angiopoietin-2 expression similar to effects that we have observed.

We also found that intercellular adhesion molecule-1 (ICAM1) and mucin 1 (MUC1) gene expressions of small AE cells were increased in coculture with both types of mesenchymal stem cells. ICAM1 is highly expressed by most epithelial cells, including those of the lung, and expression has been demonstrated to be strongly increased by the stimulation with inflammatory-relevant cytokines such as interferon-gamma, interleukin-1-beta, or tumor necrosis factor-alpha [18, 39]. Thus, the observed increased gene expression of AE cells can be likely attributed to the secretion of various cytokines from mesenchymal stem cells. In general, ICAM1 expression is increased in response to inflammatory agents and various pathological conditions: tobacco smokers, patients with chronic airflow limitation, and organic dust lead to upregulation of ICAM1 expression [19]. In the setting of lung ischemia-reperfusion injury, ICAM 1 expression had a biphasic pattern of early downregulation and a late-phase upregulation [40]. Similar to ICAM1, MUC1 expression is also affected by pathological conditions. Smokers have an increased frequency of mucin-secreting cells [41]. The induced secretion of mucins has been in general associated with anti-inflammatory actions, including those in response to toxic environmental agents [42] or viruses [43, 44]. Also, MUC1 has been shown to provide protection from ischemia-reperfusion injury in renal epithelial cells [45]. Both, ICAM 1 and MUC1, have a complex role in the setting of epithelial cell damage. To determine whether the observed net effect of cocultures may be positive or negative in terms of reducing the inflammatory response probably requires moving into more clinically oriented models.

## 5. Conclusions

MSCs and their secreted factors have been shown to influence AE cells positively. Their mode of action is complex, increasing viability and affecting gene expression of AE cells. As some effects seem to be specific for the individual tissue origin of mesenchymal stem cells, their organ source needs to be taken into consideration for prospective clinical applications.

## Figures and Tables

**Table 1:** Gene expression analyses of cultured airway epithelial (AE) cells. Gene expression was measured using quantitative real-time PCR. AE cells were cultured for three days either alone or in coculture with mesenchymal stem cells derived from the bone marrow (BM) or adipose tissue (AD). The expression of intercellular adhesion molecule 1 (ICAM1), aquaporin 5 (AQP5), gap junction alpha-1 protein (GJA1), tubulin alpha-1A (TUBA1A), secretoglobin family 1A member 1 (SCGB1A1), and mucin 1 (MUC1) was quantified using real-time PCR. Data were normalized to expression of AE controls; total human lung RNA was used as positive control. Data are given as means ± standard deviation. ∗ indicates statistically significant difference between control and coculture.

	ICAM1	AQP5	GJA1	TUBA1A	SCGB1A1	MUC1
AE	1.00	1.00	1.00	1.00	1.00	1.00
AE-BM	1.81^∗^ ± 0.19	2.49 ± 1.67	1.10 ± 0.17	1.16 ± 0.37	2.17 ± 1.33	4.24^∗^ ± 2.12
AE-AD	1.96^∗^ ± 0.25	2.99 ± 1.86	1.19 ± 0.11	1.35 ± 0.25	2.92 ± 1.98	4.04^∗^ ± 1.74
Lung	32.6 ± 26.3	95.9 ± 91.3	2.89 ± 0.20	4.10 ± 1.15	11,006.8 ± 10,674.2	22.5 ± 16.5

**Table 2:** Protein concentration analyses of cultured airway epithelial (AE) cells. The concentrations of coagulation factor 3 and angiopoietin 2 in cell culture media were measured using ELISAs. AE cells were cultured for three days either alone or in coculture with mesenchymal stem cells (MSCs) in inserts. Mesenchymal stem cells were obtained from the bone marrow (BM) or adipose tissue (AD). Concentrations were measured in culture medium that had no addition of any cells and were used subsequently as blank control and subtracted from all other measurements, which are given as blanked protein concentrations in pg/mL. Data are given as means ± standard deviation. ∗ indicates statistically significant difference between AE culture alone and coculture.

	Coagulation factor 3	Angiopoietin 2
Medium blank	2.2 ± 0.4	51.9 ± 0.8
BM MSCs	0.0 ± 0.5	−4.6 ± 2.7
AD MSCs	3.2 ± 4.7	2.4 ± 7.8
AE	600.5 ± 182.6	−15.8 ± 1.6
Total AE in BM MSC coculture	612.6 ± 154.7	−17.1 ± 4.6
Net AE in BM MSC coculture	612.6 ± 154.8	−12.5 ± 3.5
Total AE in AD MSC coculture	553.3 ± 120.8	1.5 ± 7.5
Net AE in AD MSC coculture	550.1 ± 120.9	−0.9^∗^ ± 4.4

## Data Availability

All data are given in the manuscript. Raw data are available from the authors upon request.
